# Methylation status of insulin-like growth factor-binding protein 7 concurs with the malignance of oral tongue cancer

**DOI:** 10.1186/s13046-015-0138-5

**Published:** 2015-02-24

**Authors:** Li-Hsuen Chen, Dai-Wei Liu, Junn-Liang Chang, Peir-Rong Chen, Lee-Ping Hsu, Hon-Yi Lin, Yu-Fu Chou, Chia-Fong Lee, Miao-Chun Yang, Yu-Hsuan Wen, Wen-Lin Hsu, Ching-Feng Weng

**Affiliations:** Department of Life Science and the Institute of Biotechnology, National Dong Hwa University, Hualien, Taiwan; Department of Radiation Oncology, Buddhist Tzu Chi General Hospital, Hualien, Taiwan; School of Medicine, Tzu Chi University, Hualien, Taiwan; Department of Pathology and Laboratory Medicine, Taoyuan Armed Forces General Hospital, Taoyuan, Taiwan; Department of Biomedical Engineering, Ming Chuan University, Taoyuan, Taiwan; Department of Otolaryngology, Buddhist Tzu Chi General Hospital, Hualien, Taiwan; Department of Otolaryngology, Buddhist Taichung Tzu Chi Hospital, Taichung, Taiwan; Department of Radiation Oncology, Buddhist Dalin Tzu Chi Hospital, Chia-Yi, Taiwan

**Keywords:** IGFBP-7, Head and neck cancer, Invasion, EMT

## Abstract

**Background:**

Aberrant insulin-like growth factor-binding protein 7 (IGFBP-7) expression has been found in various cancers such as prostate, breast, and colon. IGFBP-7 induced the apoptosis of tumor and potentially predicted the clinical outcome in some cancers is further demonstrated. This study investigates the causes and underlying mechanisms of aberrant IGFBP-7 expression in unravelling head and neck squamous cell carcinoma (HNSCC).

**Methods:**

A total of 47 oral tongue cancer patient samples were primarily analyzed for the methylation status in 5′ region of IGFBP-7 by methylation-specific PCR (MS-PCR). Subsequently the invasion, overexpression, and knockdown of IGFBP-7 in the HNSCC A253 invasive subpopulation were employed to examine the effect of IGFBP-7. The epithelial–mesenchymal transition (EMT) marker genes and AKT/GSK3β/β-catenin signaling were further evaluated by Western blot for the understanding the role of aberrant IGFBP-7 expression and thereof putative mechanism.

**Results:**

EMT expressed in the invasive subpopulation of HNSCC cell lines (A253 and RPMI 2650) was contemporary with the down-regulation of IGFBP-7. After treatment with 5-AZA-2′ deoxycytidine, the de-methylated CpG sites in the 5′ region of IGFBP-7 were observed and IGFBP-7 mRNA expression was also restored. Accordingly, re-expression IGFBP-7 in invasive subpopulation of A253 could induce the mesenchymal–epithelial transition (MET) and concurrently inhibited the cell invasion. Moreover, IGFBP-7 methylation status of 47 oral tongue tumors showed a positive correlation to invasive depth of the tumor, loco-regional recurrence, and cancer sequence.

**Conclusions:**

IGFBP-7 can alter EMT relative marker genes and suppress cell invasion in A253 cell through AKT/GSK3β/β-catenin signaling. The epigenetic control of IGFBP-7 in the invasion and metastasis of HNSCC was reported, suggesting that IGFBP-7 could be a prognostic factor for the probability of invasion and a therapeutic remedy.

**Electronic supplementary material:**

The online version of this article (doi:10.1186/s13046-015-0138-5) contains supplementary material, which is available to authorized users.

## Background

Cancer of the head and neck can be categorized by the area where it begins including the oral cavity, pharynx, larynx, paranasal sinuses, and nasal cavity [1]. Head and neck cancer is the sixth leading cancer by incidence worldwide [2] and the most important risk factors for head and neck squamous cell carcinoma (HNSCC) are alcohol and tobacco addiction [3]. Treatment of HNSCC includes surgery, radiation therapy, chemotherapy, targeted therapy, or a combination of treatments. Unfortunately, the outcome of therapy in five years of overall survival for the advanced stages of HNSCC is about 50% and patients often suffer recurrences at the primary site or distant metastases [4]. Hence, the related target molecule as a biomarker for HNSCC is crucial and urgent for the strategy of clinical treatment.

Insulin-like growth factor-binding proteins (IGFBPs) are one component of the insulin-like growth factor (IGF) system. IGFBP-7 belongs to the IGFBP superfamily, also known as IGFBP-related protein 1 (IGFBP-rP1) or mac 25, and is a member of the soluble protein family that binds to IGFs with a low affinity [5]. IGFBP-7 is broadly expressed in various tissues and organs, including the brain, lung, prostate, bladder, liver, and colon [6]. The expression of IGFBP-7 is diminished in various types of human cancer cell lines, including prostate, breast, colon, and lung cancer [7-10]. Moreover, the expression of IGFBP-7 is detected in a favorable prognosis of breast cancer [9]. *In vitro* studies have been demonstrated that IGFBP-7 can induce apoptosis in many cancer cells [7,11], e.g., breast and prostate cancer cells; and acts as a potential tumor suppressor against colorectal carcinogenesis. Thereof, aberrant IGFBP-7 expression may contribute to the biological behavior of tumors and the outcomes of clinical. One report showed that recombinant IGFBP-7 (rIGFBP-7) can efficiently prompt apoptosis in human melanoma cell lines [12]. Thus, IGFBP-7 may be an efficacious anticancer agent and related experiments have provided the evidence that IGFBPs possess both IGF-dependent and -independent anti-tumor actions [7].

CpG islands (CpG-rich sequences), generally unmethylated status in normal cells, are typically existing in the promoter regions or exons of genes [13]. Aberrant DNA methylation in CpG islands of tumor suppressor genes is validated as a frequent molecular event in human carcinomas [14]. Moreover, the hyper-methylation of cytosine in CpG islands is concomitant with the suppressed initiation of gene transcription and leads to carcinogenesis [15]. In one recent study, aberrant hyper-methylation of tumor suppressor genes was demonstrated to contribute the oral carcinogenesis [16]. Additionally, the hyper-methylation of certain genes (CDKN2A, CDH13, FHIT, WWOX, CDH1, and RASSF1A) in various carcinomas such as lung cancer and HNSCC has been found to cause poor progression [17-19].

Tumor metastasis is a process that involved a series complicated deregulation of cell adhesion, extracellular matrix integrity, survival, angiogenesis, lymphangiogenesis, and cell migration [20]. One of most relevant changes that occurred during metastasis in a large number of carcinomas is through the epithelial-mesenchymal transition (EMT). Some criteria have pointed out for EMT both *in vivo* and *in vitro* [21] including a) acquisition of N-cadherin, Vimentin, beta-catenin, or transcription factors (Snail, Slug and Twist); and b) loss of E-cadherin (E-cad) or cytokeratin. One of the key molecules in the process of EMT is E-cad and the transition of phenotypes commonly involved in the down-regulation of E-cad. E-cad functionally inactivated by different mechanistic events that comprise somatic mutation, transcriptional repression, and promoter methylation. Restoring E-cad in tongue squamous cell carcinoma through the suppression of the EMT was recently reported [22]. Vimentin, the type III microfilaments (IFs) had gained much importance as a canonical marker of EMT [23] and served as a potential biomarker for predicting metastasis in malignant melanoma [24]. The molecules which can regulate EMT like Snail [25] are broadly investigated in cancer research. Previously the potential role of IGFBP-7 in cancer invasion has been reported [26]. Moreover, IGFBP-7 is silenced by DNA methylation in colorectal and gastric cancers [27,28]. To our best knowledge, the methylation status of IGFBP-7 in HNSCC has yet to be elucidated particularly in invasion. In this study, the invasive subpopulations of HNSCC cell lines (A253-3, −5, and RPMI 2650–8) were performed to investigate the unravelling causes and underlying mechanisms of aberrant in HNSCC. Our data showed that the presence of EMT in invasive subpopulations and the epigenetic control of the IGFBP-7 expression. To further confirm the role of IGFBP-7 in metastasis of HNSCC, the effect of re-expression and knockdown of IGFBP-7 in HNSCC cell line were examined. Moreover, clinical oral tongue samples were also collected and analyzed to verify a correlation between the methyl status of IGFBP-7 and the clinical outcomes of HNSCC.

## Materials and methods

### The ethical consideration

All human experiments were conducted in accordance with the Helsinki Declaration of 1975, as revised in 2000. Before experiments were conducted, all samples and clinical information were decoded and renumbered with a serial number. The followed procedures have also been approved by the Institution Review Board (IRB) of the Buddhist Tzu Chi General Hospital (Number IRB 100–101).

### Cell lines and clinical tissue sampling

Two HNSCC cell lines, A253 and RPMI 2650, were obtained from the American Type Culture Collection (ATCC). A253 maintained in McCoy's 5a Medium, RPMI 2650 maintained in Eagle's Minimum Essential Medium, both supplemented with 10% FBS and a 37°C/5% CO_2_ atmosphere. The invasive subclones of HNSCC cell lines were obtained following the method described in previous study [29] with minor modification. In brief, 100 μl of 80 μg Matrigel (cat 354234, BD) was placed on the upper chamber of the Transwell (24 well, 8 μm pore size, BD) and incubated for one hour at 37°C. The cells were then seeded on the Matrigel coated Transwell without serum and 500 μl of complete medium was added into the lower chamber. Cells were cultured for several days until cells invaded through the membrane. Cells remained on the upper chamber were wiped out using sterilized cotton swabs and the subclone of invasive cells was harvested by trypsinizing. The subclones were expanded and repeated this process. The number following cell names indicated how many times the cells were selected (A253-3: selected for 3 times, A253-5: selected for 5 times, RPMI 2650–8: selected for 8 times).

A total of 47 oral tongue cancer samples were used for testing the methylation status of the 5′ region of IGFBP-7 by methylation-specific PCR (MS-PCR). All tumor samples were dissected and stored in RNAlater at the time of radical surgery. After the IRB’s approval, the samples were re-sectioned and applied in this study. The correlation of clinical outcomes and IGFBP-7 methylation were analyzed.

### 5-AZA-2′ deoxycytidine treatment

To analyze the demethylation effect and restoration of IGFBP-7 expression, HNSCC cells were treated with 2 μM of 5-aza-2′-deoxycytidine (5′AZA) (Sigma, St Louis, MO) for 96 h and replaced the medium with 5′AZA every 48 h. Cells were washed by PBS and DNA and RNA were extracted by following methods.

### DNA extraction, bisulfite modification, MS-PCR and bisulfite genomic sequencing

The genomic DNA was isolated from cell lines and tissue samples using QIAamp DNA Mini Kit (QIAGEN). Two micrograms of genomic DNA was applied to bisulfite conversion and purified by EpiTect Fast Bisulfite Conversion Kit (QIAGEN) according to the manufacturer's instructions. MS-PCR and bisulfite genomic sequencing (BGS) were performed as previously described [30]. A total of 80 ng of bisulfite-modified DNA were used in MS-PCR and BGS.

The methylated and unmethylated specific-PCR conditions for IGFBP-7 were performed as denature at 95°C for 15 min followed by 35 cycles at 95°C for 30 sec, 60°C for 30 sec, 72°C for 30 sec and final elongation at 72°C for 5 min. The PCR for the methylated and unmethylated products were visualized and photographed on 2% agarose gel. For BGS, PCR product was purified and cloned into pCR 2.1 using TA Cloning Kit (Invitrogen). For each PCR product, five clones were sequenced and 55 CpG sites were included into this analysis. The primer sequences and PCR product sizes were showed in Additional file 1: Table S1.

### RNA extraction, cDNA synthesis and quantitation PCR

Total RNA was isolated using the Trizol reagent (Invitrogen, USA) according to the manufacturer’s instructions. Total RNA was incubated with DNase I for 15 min at 37°C. One microgram of total RNA was applied into reverse transcription reaction using First Strand cDNA Synthesis Kit (Roche). One microliter of cDNA was amplified in a total reaction volume of 20 μl containing 100 nM gene specific primer and 10 μl of 2× SYBR® Green PCR Master Mix (Applied Biosystems) using StepOne Real-Time System (Applied Biosystems). The Q-PCR experiment was performed in triplicate and the mean values were used for calculations. The IGFBP-7 mRNA expression index was normalized with the reference gene GADPH using the comparative Ct method (2^-△CT^) [31].

### Transfection of IGFBP-7 expression plasmid and siRNA against IGFBP-7

Expression plasmid of human IGFBP-7 was purchased from OriGene (#SC119176). Transient IGFBP-7 expression A253-5 cells (marked as “IGFBP-7”) were obtained using Xfect® transfection reagent (Clontech) according the manufacturer’s instructions. 5 × 10^5^ of A253-5 cells were seeded in a 60 mm dish and cultured overnight. A total of 15 μg of plasmid was used for transfection procedure for each dish. A253-5 without transfection was labeled as a control (C) and cells transfected with pEGFP-N1 were marked as “Mock” in this study. For siRNA transfection, negative control siRNA and siRNA against IGFBP-7 (#s7241) were purchased from Invitrogen. Final concentration of both siRNAs at 30 nM was used in the transfection to obtain “IGFBP-7 + NC si” and IGFBP-7 + siRNA respectively.

### Western blot

Culture cells were washed with PBS and the total protein was extracted using ProteoJET Mammalian Cell Lysis Reagent (Fermentas) according to the manufacturer’s instructions. Nuclear protein fraction was prepared using Nuclear Extract Kit (Active Motif) according to the manufacturer’s instructions. In general, 20 μg total proteins were loaded onto 10% SDS-polyacrylamide gels and then transferred onto PVDF membranes after electrophoresis. The transferred membranes were blocked with StartingBlock Blocking Buffers (Thermo Scientific) for 10 min and incubated with specific primary antibody against E-cadherin (BD), Vimentin (BD), IGFBP-7, phospho T308 AKT1, AKT1, phosphor S9 GSK3β, GSK3β (Abcam), β-catenin (GeneTax), or β-Actin (Cell signaling) at 1:1000 dilutions at 4°C overnight. The membranes then were washed three times in Tris Buffered Saline with 0.1% Tween-20 (TBST). Bands were detected using a horseradish peroxidase-linked second antibody and enhanced chemiluminescence reagents, according to the manufacturer's protocol. Bands were record by film and the intensity of the band was quantified with a GS-800 calibrated densitometer (Bio-Rad), and calculated as the optical density area of bands. The data were conducted independently in triplicate.

### Invasion assay

One hundred microliter of 80 μg Matrix gel was previously placed onto the upper chamber of 24 well Transwell with 8 μm pore size at 37°C for 2 h and 5 × 10^4^ cells with 200 μl of culture medium without FBS were seeded on the gel. Five hundred microliter of complete medium was added into the lower chamber of the Transwell. After 24 h incubation, cells on the upper chamber of the Transwell were wiped out using cotton swaps and the Transwell was stained with Diff-Quik stain solution according to the manufacturer's protocol. Five images were photographed for each Transwell under 50 or 100× magnification. Cell numbers were counted and the mean values were used for calculations.

### IGFBP-7 Immunohistochemistry

Immunohistochemistry was performed using the method described previously [32] with minor modification. Four μm sections from paraffin-embedded tissues were deparaffinized and treated with 3% hydrogen peroxidase in methanol. Heat-induced epitope retrieval was achieved by incubation in 0.01 M citrate buffer (pH 6.0) and heated in a microwave oven (700 W) by two cycles of 5 min. The sections were then placed in a humidified chamber with 10% normal horse serum (Dako) at room temperature (RT) for 20 min and incubated with primary antibody against IGFBP-7 (1:20, goat polyclonal IgG, sc-6064, Santa Cruz, USA) at RT for 1 h. The sections were rinsed with PBS and incubated with an appropriate dilution of biotinylated anti-goat antibody (Dako) at RT for 1 h, rinsed with PBS, and incubated with avidin-biotin complex (Dako) at RT for 30 min. The substrate chromogen, 3% amino-9-ethylcarbazone (Dako) was developed for 5 to 8 min. The slides were then counterstained with Mayer hematoxylin and photographed. The positive control was sampled from normal oral tongue epithelium tissue known to express IGFBP-7.

### Statistical analysis

For the *in vitro* study, data are present as mean ± SD. Statistical analysis between the control and the treatment groups were compared using the Student’s t-test. Significantly different data was labeled using *, compared to “Mock”, and # compared to the negative control siRNA group, respectively. A Single mark represents p < 0.05, double marks represent *p* < 0.01, and triple marks represent *p* < 0.001. Survival data were calculated according to the Kaplan-Meier method and were compared using the log-rank test. Statistical analysis was performed using StatView (version 5.0; SAS Institute, Cary, NC). A *p* value < 0.05 was considered statistically significant.

## Results

### Invasive subclones of HNSCC cell present EMT and down-regulation of IGFBP-7

**Figure 1:**
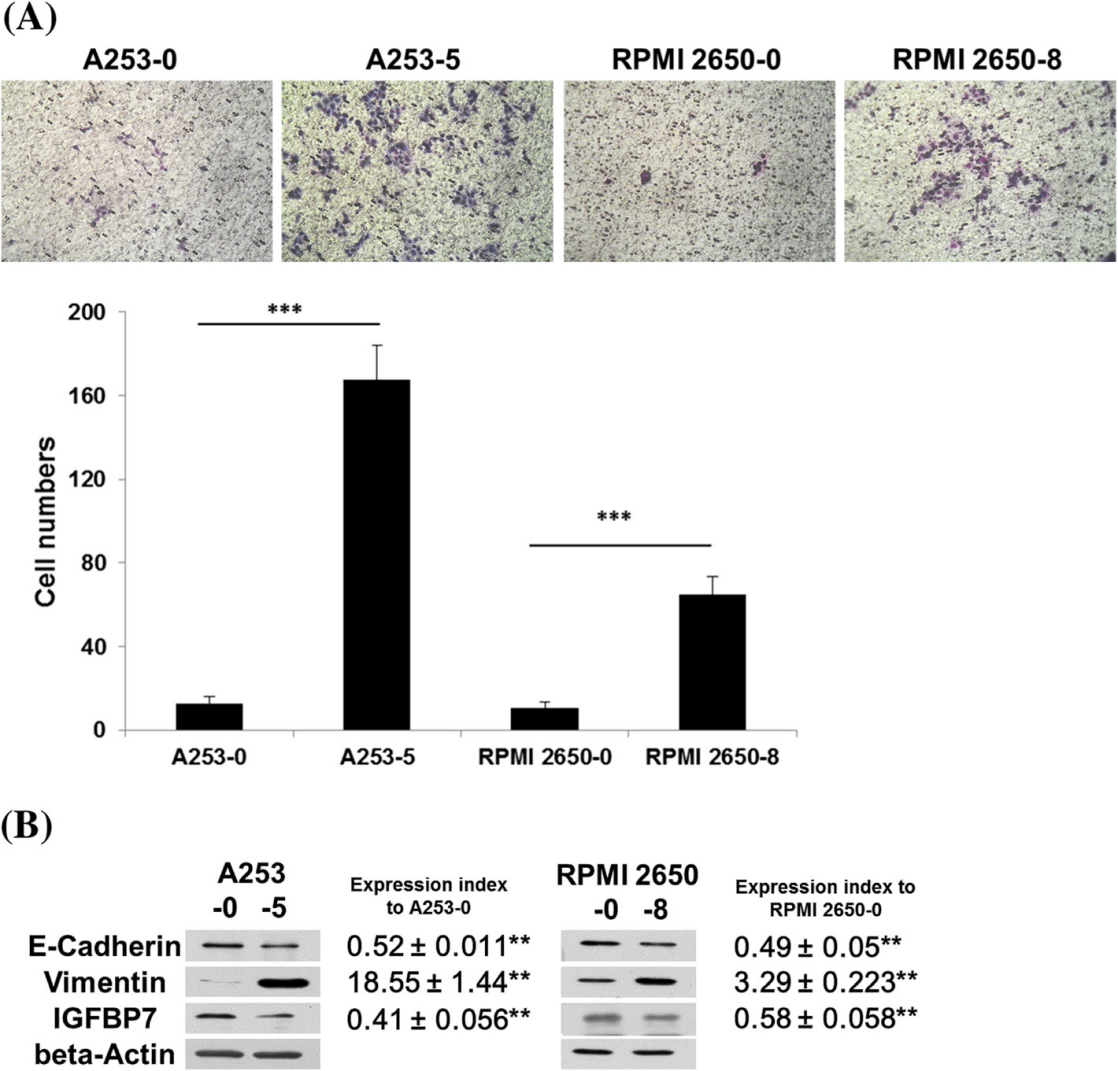
**Characterizations of invasive subpopulation of HNSCC cell lines. (A)** The invasion assay of A253, RPMI 2650 and their invasive subpopulations. Images show that the cells invaded through the Matrigel coated membrane at a magnification of ×50. **(B)** Protein expression of EMT related genes and IGFBP-7 in A253, RPMI 2650 and their invasive subpopulations. Expression index was normalized with beta-actin and ratio to A253 or RPMI 2650, respectively. ** indicated *p* < 0.01; *** indicated *p* < 0.001.

Invasive subclones of HNSCC cell lines A253-5 and RPMI 2650–8 have shown significantly higher cell mobility when compared to the original cell line A253-0 and RPMI 2650–0. After 24 h incubation, only few cells (<20) could migrate in 8 μm pore size toward the lower side of membrane coated with Matrigel (Figure 1A). In Figure 1B shows that E-cadherin was down-regulated whereas Vimentin was up-regulated in A253-5 and RPMI 2650–8 cells, revealing that the EMT presents in HNSCC cell lines. Concurrently, the down-regulated IGFBP-7 in both A253-5 and RPMI 2650–8 cells was also validated.

### Methylation status of IGFBP-7 and retrieval of IGFBP-7 expression

**Figure 2:**
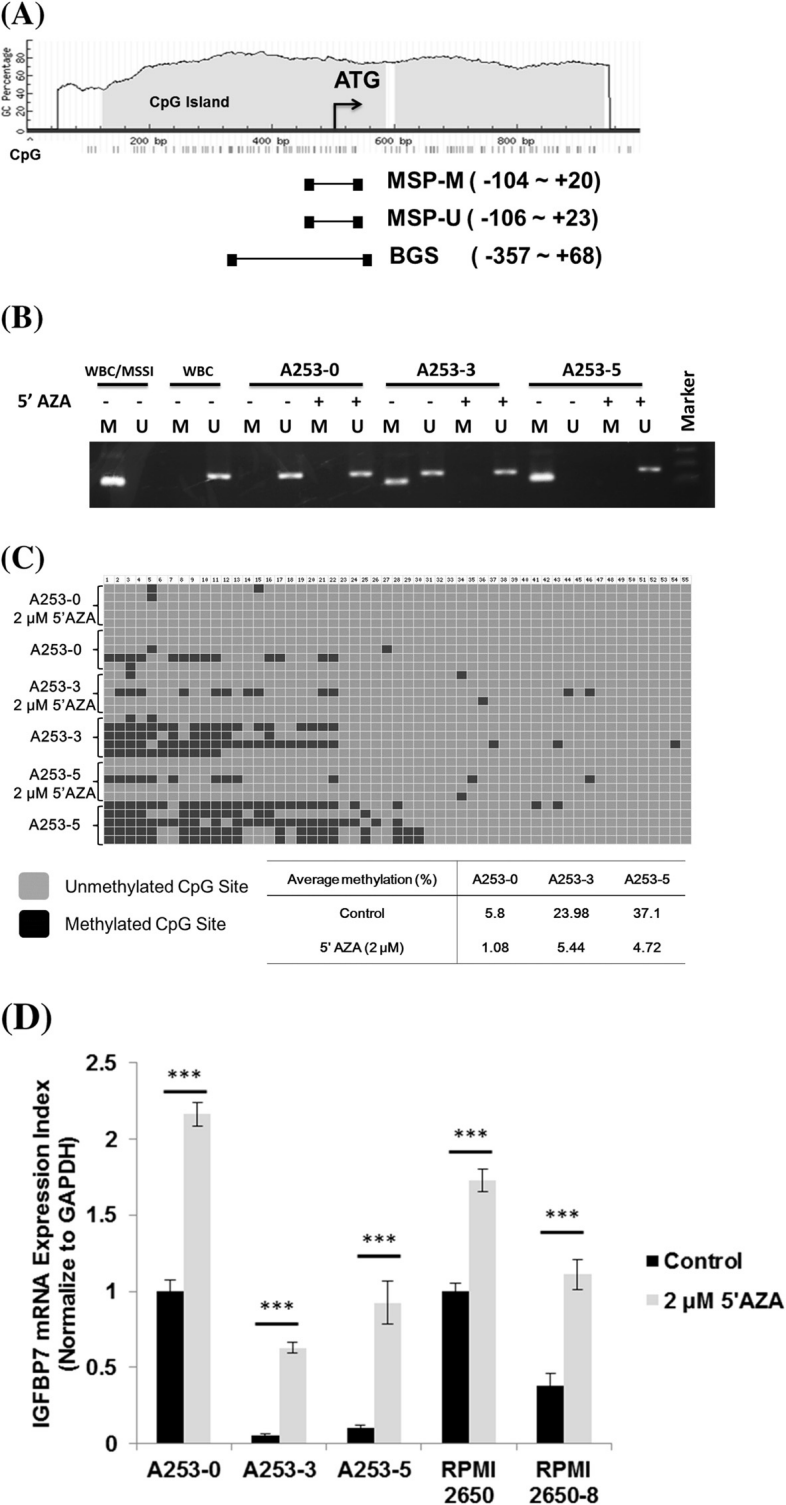
**Methylation analysis of IGFBP-7 promoter and effect of 5′AZA on HNSCC cell lines. (A)** Illustration of 5′ region of IGFBP-7. Position of CpG islands and primers for IGFBP7 methylation assay. **(B)** MSP assay for A253 and its invasive subpopulations. M indicated methylated amplicon and U indicated unmethylated amplicon. **(C)** BGS of 5′ region in IGFBP-7 for A253 and its invasive subpopulations. Data are shown with an average methylation percentage with or without 5′AZA treatment for A253 cells. **(D)** Restored IGFBP7 mRNA expression after 5′AZA treatment. IGFBP-7 mRNA expression was detect using Q-PCR after treatment with or without 5′AZA in HNSCC cell lines. *** indicated *p* < 0.001.

Methylation status in the 5′ region of IGFBP-7 was examined by MS-PCR and BGS. In Figure 2A shows that −500 to +500 sequences of transcription start site of IGFBP-7 were analyzed. Additional file 1: Table S1 lists the CpG islands covering transcription start site was used to design primers and primer sets. We found the 5′ region of IGFBP-7 might be partially methylated because 124 bp amplicon of methylated specific PCR (M) was presented in all A253 cells and abrogated after 5′AZA treatment (Figure 2B). Alongside, genomic DNA from normal human white blood cell (WBC) was extracted and treated with or without CpG Methyltransferase (M.SssI) in MS-PCR to serve as positive and negative controls, respectively.

The methylation status of 55 CpG sites in the 5′ region of IGFBP-7 was determined by bisulfite sequencing of the A253 cells. In Figure 2C shows the average methylated CpG sites from 5 clones of A253 cells that were analyzed. The original A253-0 cell presented only 5.8% methylated CpG sites of the 5′ region in IGFBP-7 whereas the invasive subclones A253-3 and A253-5 showed around 24-37% methylated CpG sites of the 5′ region in IGFBP-7. Interestingly, the treatment of 5′AZA led to the unmethylated CpG sites and restore mRNA expression of IGFBP-7 in HNSCC cell lines (Figure 2D).

### Overexpression of IGFBP-7 induce MET and reduce cell invasion

**Figure 3:**
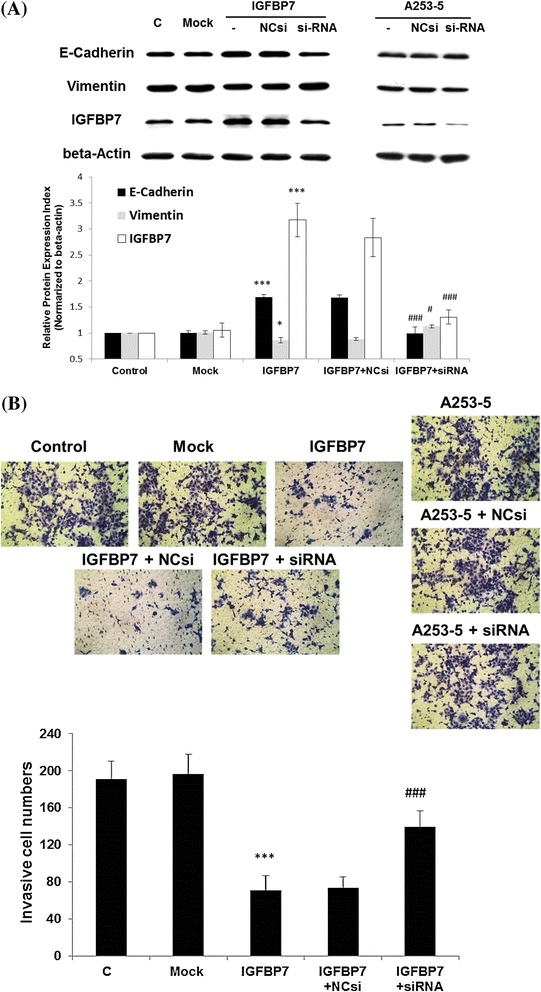
**Overexpression of IGFBP-7 in A253-5 cell reverses EMT and suppress cell invasion. (A)** Western blot results show up-regulated E-cadherin and down-regulated Vimentin after overexpression IGFBP-7 in A253-5 and reversed by siRNA knockdown. **(B)** Invasion assay after overexpression of IGFBP-7 in A253-5 cell. Images show that the cells invaded through the Matrigel coated membrane at a magnification of × 100. C: A253-5, Mock: A253-5 transfected with pEGFP-N1, IGFBP7: Transient IGFBP-7 expression A253-5 cells, IGFBP7 + NCsi: Transient IGFBP-7 expression A253-5 cells transfected with negative control siRNA, IGFBP7 + siRNA: Transient IGFBP-7 expression A253-5 cells transfected with siRNA against IGFBP7. * indicated *p* < 0.05, ** indicated *p* < 0.01, *** indicated *p* < 0.001 compared with A253-5 Mock cells. # indicated *p* < 0.01, ### indicated *p* < 0.001 compared with IGFBP7 + NCsi.

As we know the treatment of 5′AZA can cause the universal demethylation effect in all methylated genes. To investigate the specific effect of IGFBP-7 on invasive subclone of HNSCC, the expression plasmid of IGFBP-7 was transfected to A253-5 cells. After 24 h transfection, the protein level of IGFBP-7 was 3 fold higher when compared to the Mock, which indicated the success of transfection (Figure 3A). Interestingly, E-cad was also increased and contrarily Vimentin was decreased in while IGFBP-7 was overexpressed. The change of these two marker genes in overexpression of IGFBP-7 may infer the occurrence of MET. Moreover, knockdown of IGFBP-7 using siRNA technique in re-expression A253-5 cells showed the reversion of MET. This result further suggested that IGFBP-7 might alter the expression of EMT-relative genes.

These transfected cells were also evaluated the invasive ability using the invasion assay. In Figure 3B shows that the re-expression of IGFBP-7 in A253-5 cells revealed dramatic repression of cell invasion when compared with the Mock cells (*p* < 0.001). After the transfection with siRNA to knockdown the expression of IGFBP-7, the ability of invasion was restored when compared with the negative control siRNA (*p* < 0.001).

### Involvement of AKT-GSK3β pathway in IGFBP-7 induced MET

**Figure 4:**
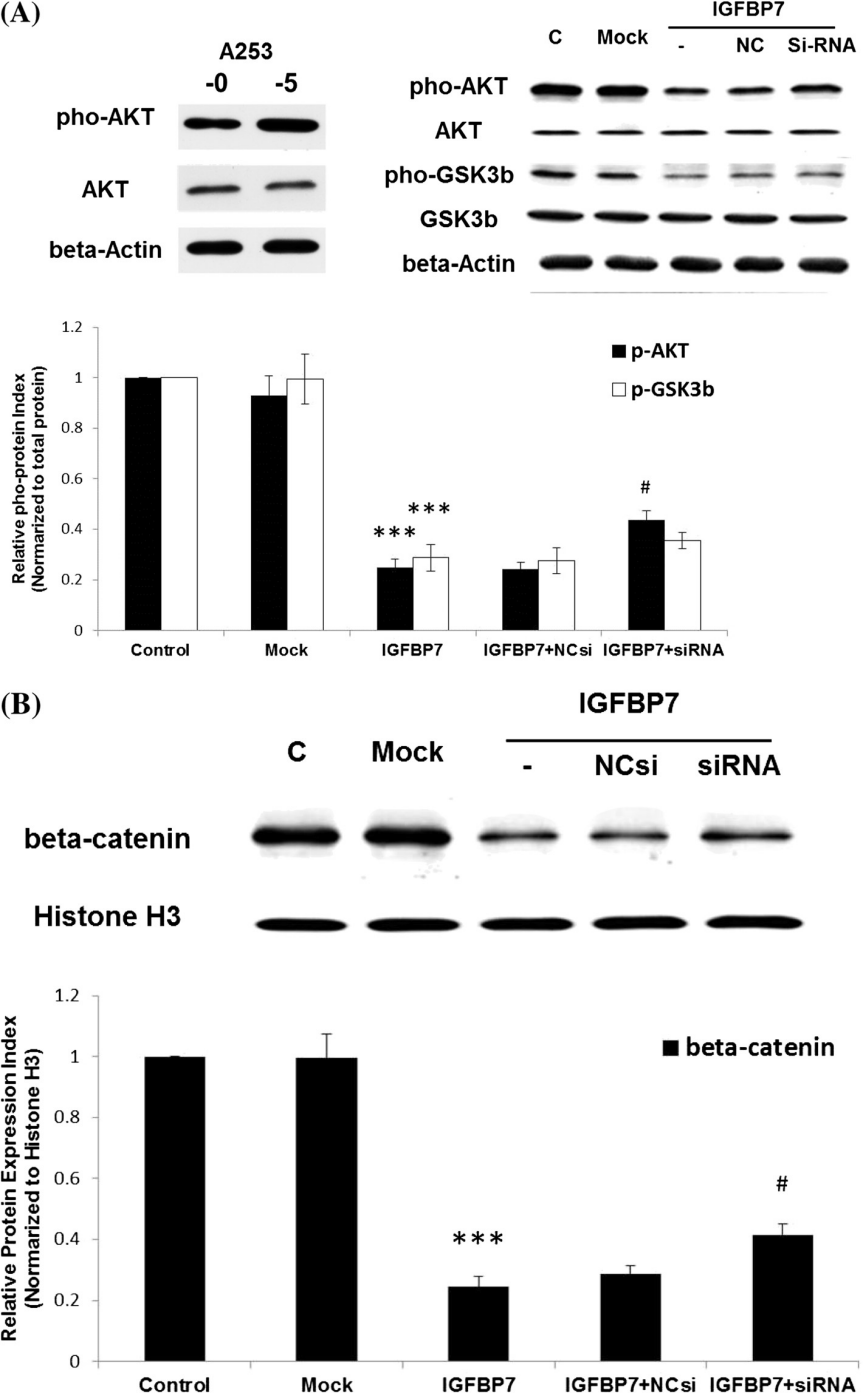
**Inactivate of AKT/GSK3β and reduce translocation of β-catenin in IGFBP-7 overexpression A253-5 cells. (A)** Western blot analyzed the phosphorylation of AKT (Thr 308) and GSK3β (Ser 9). **(B)** Western blot using protein from nuclear fraction is analyzed. C: A253-5, Mock: A253-5 transfected with pEGFP-N1, IGFBP7: Transient IGFBP-7 expression A253-5 cells, IGFBP7 + NCsi: Transient IGFBP-7 expression A253-5 cells transfected with negative control siRNA, IGFBP7 + siRNA: Transient IGFBP-7 expression A253-5 cells transfected with siRNA against IGFBP7. *** indicated *p* < 0.001 compared with A253-5 Mock cells. # indicated *p* < 0.01 compared with IGFBP7 + NCsi.

The AKT signaling is an important pathway in the EMT [33-35]. We also found the phosphorylation of AKT was higher in A253-5 when compared to A253 cells (Figure 4A). Subsequently, the AKT and its downstream signaling molecules were examined to understand the underlying mechanism of IGFBP-7 induced MET. In Figure 4A shows that persistently activated AKT phosphorylation and inhibited GSK3β cause the nuclear translocation of β-catenin (Figure 4B). After re-expression of IGFBP-7 in A253-5 cells, the phosphorylated AKT and GSK3β were down-regulated that consequently caused less translocation of β-catenin when compared to the Mock cells (*p* < 0.001). Moreover, the inhibition of AKT/GSK3β/β-catenin signaling might be partially re-activated by transfection of siRNA against IGFBP-7.

### Clinical Studies

The expression of IGFBP-7 in oral tongue tumor lesions is validated by immunohistochemical staining. Additional file 2: Figure S1 shows the normal epithelium of oral tongue tissue expresses IGFBP-7. The pathologic stage of the IGFBP7 IHC positive case is T2N2bM0. Compared with different AJCC (American Joint Committee on Cancer) stage of tumor, the advance tumor lesions (T4aN2bM0) showed the absence of IGFBP-7 expression. Subsequently, 47 oral tongue tumor lesions were also performed to analyze the IGFBP-7 methylation status by MS-MSP. There were 31 methylated and 16 unmethylated in 5′ region of IGFBP-7 (Table 1). Table 1 illustrates the clinic-pathological factors related to IGFBP-7 methylation status. The clinic-pathological factors are correlated to IGFBP-7 methylation status. The data indicated the methylation status of IGFBP-7 was associated with invasive depth, loco-regional recurrence and cancer sequence (*p* = 0.03, 0.011 and 0.029, respectively).

**Table 1:** **Demographic data and clinical outcome of the oral tongue patients**

	**IGFBP7**		
	**Methylation**	**Unmenthylation**	**p.value**
**Gender**			0.444
Male	22	13	
Female	9	3	
**Age at Diagnosis**	54.3 ± 10.7	50.9 ± 7.6	0.267
**Tumor Size (mm)**	30.2 ± 13.8	27.1 ± 11.5	0.451
**Invasion Depth (mm)**	17.3 ± 10.3	9.7 ± 6.4	0.030*
**AJCC Stage**			0.437
Early (I-II)	10	7	
Locally Advanced (III-IVB)	21	9	
**Perineural Infiltration**			0.458
Yes	19	8	
No	12	8	
**Lymphovascular Permeation**			0.458
Yes	19	8	
No	12	8	
**Extracapsular Spread**			0.444
Yes	9	3	
No	22	13	
**Postoperative Radiotherapy**			0.236
Yes	21	8	
No	10	8	
**Adjuvant Chemotherapy**			0.357
Yes	14	5	
No	17	11	
**Loco-regional Recurrence**			0.011*
Yes	13	1	
No	18	15	
**Distant Metastasis**			0.133
Yes	4	12	
No	27	4	
**Cancer Sequence**			0.029*
First Cancer	20	15	
Second Primary	11	1	

Data are expressed as mean ± SD. *p < 0.05.

**Figure 5:**
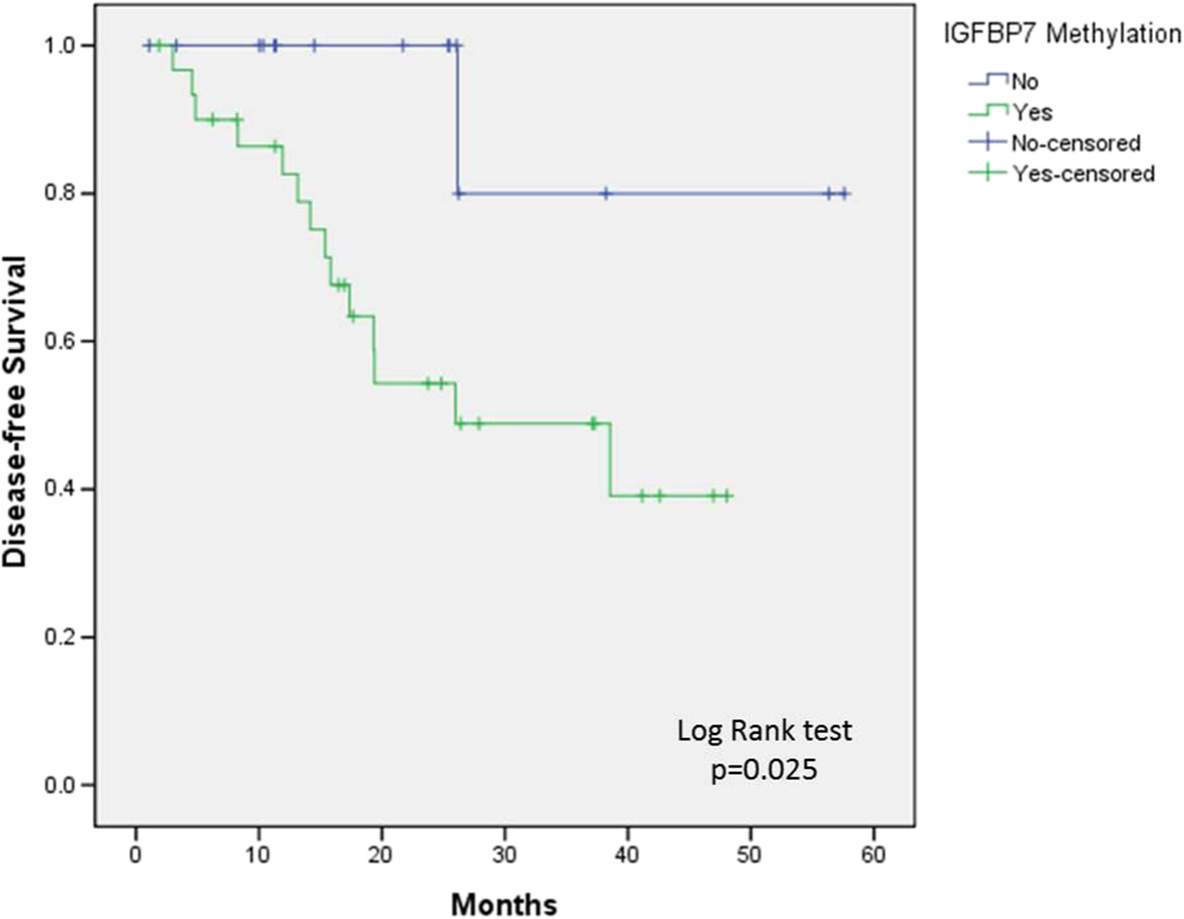
**Survival analysis of oral tongue patients with methylation status of IGFBP-7 promoter region.**

The disease-free survival (DFS) in patients with methylated IGFBP-7 (1/2/4 year: 85%/ 58%/ 40%) was significantly poorer than those of patients with unmethylated IGFBP-7 (1/2/4 year: 100%/ 100%/ 80%) (*p* = 0.025) (Figure 5). Furthermore, univariate and multivariate analyses showed that the AJCC stage of tumor and methylation status of IGFBP-7 was significantly correlated with DFS (Additional file 3: Table S2).

## Discussion

The insulin-like growth factor (IGF) pathway is involved in many cellular progression including proliferation, differentiation, cell survival, tumor invasion and metastasis, and inhibition of apoptosis [36,37]. Circulating IGFs are binding to high affinity IGF binding proteins (IGFBP-1 to 6) to maintain IGFs bioactivity in extracellular fluids. Recently, there are numerous studies demonstrated the IGF-independent actions of IGFBPs. Especially, IGFBP-3 [38] and IGFBP-5 [39] show their tumor suppressor character in HNSCC. In the present study provides first evidence the down-regulation of IGFBP-7 by hyper-methylation in HNSCC cell lines. The data show that the invasive phenotype of subpopulation in the HNSCC cell lines is accompanied by the down-regulation of IGFBP-7. This scenario was also validated in 47 oral tongue tumor lesions by analyzing IGFBP-7 expression and its promoter methylation status. Taken together, the results suggest that the decreased expression of IGFBP-7 in HNSCC is regulated by DNA methylation. Although some reports demonstrate the anti-tumor effects of IGFBP-7 including cell growth inhibition [7,40], senescence [12,41,42], and apoptosis [12,42,43], our study is evident that the down-regulation of IGFBP-7 in invasive HNSCC cell lines which show EMT feature. In pervious report has suggested the potential role of IGFBP-7 in cancer invasion [26,44]. Aberrant hyper-methylation of tumor suppressor genes are well known to contribute to oral carcinogenesis [16]. Moreover, adenovirus expressing IGFBP7 has proof it anti-metastasis effect on Hepatocellular carcinoma by xenograft models [45]. Taken all known information into considerations, the expression of IGFBP-7 seems to play a vital role involved in the invasion and metastasis of HNSCC.

Demethylating agents are used to result in the hypo-methylation of DNA by inhibiting DNA methyltransferase, such as 5-aza-2′-deoxycytidine (5′AZA), which is also chose to test its effect in the present work. Treated A253 cells with 5′AZA can inhibit IGFBP-7 promoter methylation and renovate the expression of IGFBP-7 but might also cause the re-expression of other tumor suppressor genes since 5′AZA is considered as an anti-tumor agent [46]. Next, the over-expression of IGFBP-7 in invasive A253-5 cell is employed to study the anti-invasive effect of IGFBP-7. Our results indicated dramatic (*p* < 0.001) repression of cell invasion in the re-expression of IGFBP-7 in A253-5 cells. When siRNA was employed to knockdown the expression of IGFBP-7, the ability of invasion was significantly (*p* < 0.001) restored. These results reveal that IGFBP-7 plays a pivotal role of invasion control in HNSCC.

The up-regulation of E-cad, down-regulation of Vimentin, and inhibition of cell growth and invasiveness, suggesting the presence of MET in IGFBP-7 overexpressed A253-5 cells. Additionally, the inactivation of AKT is also noted in IGFBP-7 overexpressed A253-5 cells. In general, AKT modulates numerous processes characteristic of cancer including cell survival, cell cycle regulation and apoptosis [47-49]. Activated AKT can also induce EMT and increase the invasiveness of squamous cell carcinoma [33]. AKT-induced EMT is reported through the up-regulated expression of Snail which is one of the E-cad transcriptional repressors [50,51]. The involvement of another important pathway GSK3β/β-catenin axis in AKT-induced EMT is demonstrated [34]. GSK3β, one of AKT substrate, can inhibit its activity by serine phosphorylation (Ser9). Firstly, the inhibition of GSK3β in turn stabilizes Snail from the degradation [52]. Secondly, the phosphorylation of GSK3β at Ser9 can thwart the phosphorylating β-catenin and stabilize β-catenin in the cytoplasm [53]. After β-catenin accumulation, there was an increase in the transcriptional activity while it translocated from cytoplasm into the nucleus for binding transcriptional factors like T-cell factor/lymphoid enhancer factor family (TCF/LEF) [54]. The numbers of cellular oncogenes such as c-MYC, cyclin D1, and the EMT-related genes Slug and Vimentin are also regulated in this fashion [55-57].

Recent study in searching prognostic marker of HNSCC, tissue microarrays were performed to show that Ki67 expression was associated with worse prognosis and lymph node metastasis [58]. A meta-data analysis implies that aldehyde dehydrogenase 1 (ALDH1)-positive patients had worse prognosis including the decrease of overall survival and disease-free survival [59]. SIRT6 and SIRT7 were altered in peripheral blood leukocytes of HNSCC, implying they are potential circulating prognostic markers for HNSCC [60]. Furthermore, parathyroid hormone-like hormone (PTHLH) which encodes parathyroid hormone-related protein (PTHrP) could play a character in the pathogenesis of oral squamous cell carcinoma by interfering cell proliferation and cell cycle, and the protein levels of PTHrP might potentially serve as a prognostic indicator for the evaluating patients with HNSCC [61]. As herein we report that IGFBP-7 may serve a biomarker for prognosis of HNSCC, the *in vitro* studies also reveal the underline mechanism of IGFBP-7 involved in invasion of HNSCC.

## Conclusion

We report that IGFBP-7 can alter EMT relative marker genes and suppress cell invasion in A253 cell through the inhibition of AKT activation. Aberrant hyper-methylation of IGFBP-7 in oral tongue tumors was significantly concomitant with tumor progression (invasive depth, loco-regional recurrence, and cancer consequence) and poor prognosis. These results suggest that IGFBP-7 plays a potential role in regulating malignant behavior of head and neck squamous cell carcinoma and may be a novel therapeutic target for determination in the progression of HNSCC patients.

## Additional files

Additional file 1: Table S1. Supporting Information Table S1 PCR primers used in this study. Additional file 2: Figure S1. Immunohistochemistry of IGFBP-7 in oral tongue specimen (A, B, and C). Black arrow shows IGFBP-7 expression in oral tongue specimen. Histology of oral tongue specimen using hematoxylin and eosin stain (D, E, and F). The pathologic stage of IGFBP7 positive specimen (B and E) is T2N2bM0. The pathologic stage of IGFBP7 negative specimen (C and F) is T4aN2bM0. Images show at a magnification of × 200. Additional file 3: Table S2. Supporting Information Prognostic factors of disease-free survival in oral tongue patients.

## Abbreviations

5′AZA: 5-aza-2′-deoxycytidine
AJCC: American Joint Committee on Cancer
AKT: Protein kinase B
BGS: Bisulfite genomic sequencing
DFS: Disease-free survival
E-cad: E-cadherin
EMT: Epithelial–mesenchymal transition
FBS: Fetal bovine serum
GSK3β: Glycogen synthase kinase 3 beta
HNSCC: Head and neck squamous cell carcinoma
IGF: Insulin-like growth factor
IGFBP-7: Insulin-like growth factor-binding protein 7
MET: Mesenchymal–epithelial transition
MS-PCR: Methylation-specific polymerase chain reaction
PVDF: Polyvinylidene difluoride
Q-PCR: Quantitative polymerase chain reaction
siRNA: Small interfering RNA
