# Supplementary material for: Methylation status of insulin-like growth factor-binding protein 7 concurs with the malignance of oral tongue cancer
Source: J Exp Clin Cancer Res. 2015 Feb 24;34(1):20. doi: 10.1186/s13046-015-0138-5 (PMC4355468; doi:10.1186/s13046-015-0138-5)
Supplement: Additional file 1: Table S1. — Supporting Information Table S1 PCR primers used in this study. [file 13046_2015_138_MOESM1_ESM.pdf]

Supporting Information Table 1. PCR primers used in this study

Primer name		Sequence	Product size (bp)
**IGFBP-7-RT-PCR**	F	5'- CACTGGTGCCCAGGTGTACT -3'	240
	R	5'- TTGGATGCATGGCACTCATA -3'	
**GAPDH**	F	5’- ACCACAGTCCATGCCATCAC -3’	452
	R	5’- TCCACCACCCTGTTGCTGTA -3’	
**IGFBP-7-MSP (M)**	F	5'- GGGGTGTGGTTTTCGCGGATAGGTC -3'	124
	R	5'- CGCAACGACGACCGCTCCATAACG -3'	
**IGFBP-7-MSP (U)**	F	5'- TTGGGGTGTGGTTTTTGTGGATAGGTT -3'	129
	R	5'- ACACACAACAACAACCACTCCATAACA -3'	
**IGFBP-7-BGS**	F	5’- GGAAATGGGGAGAAATTAGA -3’	425
	R	5’- AAAAAACAAAAACAAAAACAACAAC -3’	
