# Supplementary material for: Methylation status of insulin-like growth factor-binding protein 7 concurs with the malignance of oral tongue cancer
Source: J Exp Clin Cancer Res. 2015 Feb 24;34(1):20. doi: 10.1186/s13046-015-0138-5 (PMC4355468; doi:10.1186/s13046-015-0138-5)
Supplement: Additional file 2: Figure S1. — Immunohistochemistry of IGFBP-7 in oral tongue specimen (A, B, and C). Black arrow shows IGFBP-7 expression in oral tongue specimen. Histology of oral tongue specimen using hematoxylin and eosin stain (D, E, and F). The pathologic stage of IGFBP7 positive specimen (B and E) is T2N2bM0. The pathologic stage of IGFBP7 negative specimen (C and F) is T4aN2bM0. Images show at a magnification of × 200. [file 13046_2015_138_MOESM2_ESM.pdf]

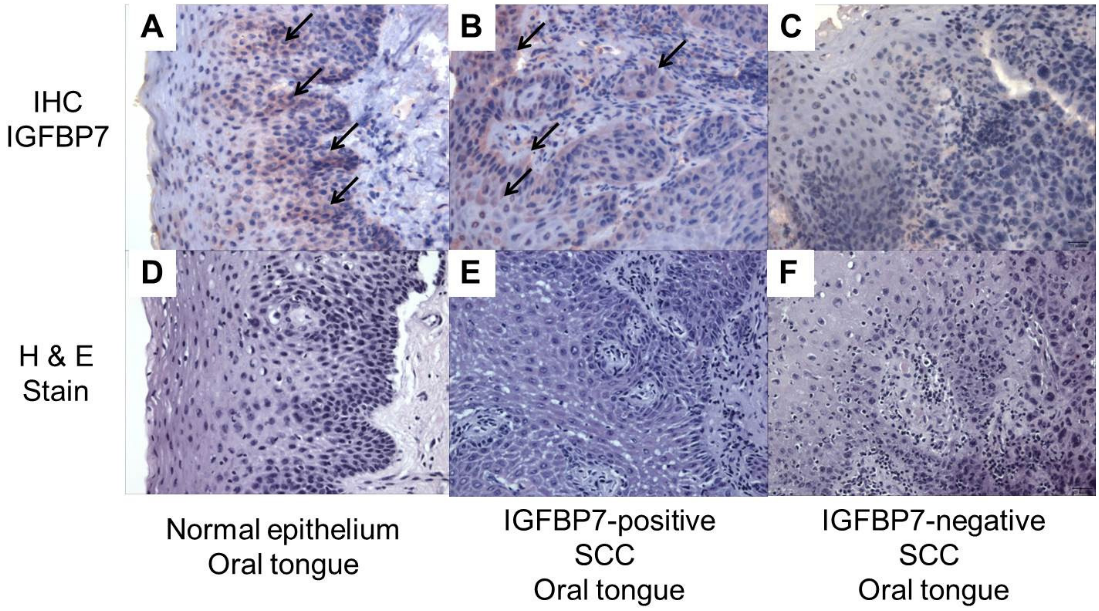

Supporting Information Figure 1. Immunohistochemistry of IGFBP-7 in oral tongue specimen (A, B, and C). Black arrow shows IGFBP-7 expression in oral tongue specimen. Histology of oral tongue specimen using hematoxylin and eosin stain (D, E, and F). The pathologic stage of IGFBP7 positive specimen (B and E) is T2N2bM0. The pathologic stage of IGFBP7 negative specimen (C and F) is T4aN2bM0. Images show at a magnification of x 200.
