# Supplementary material for: Methylation status of insulin-like growth factor-binding protein 7 concurs with the malignance of oral tongue cancer
Source: J Exp Clin Cancer Res. 2015 Feb 24;34(1):20. doi: 10.1186/s13046-015-0138-5 (PMC4355468; doi:10.1186/s13046-015-0138-5)
Supplement: Additional file 3: Table S2. — Supporting Information Prognostic factors of disease-free survival in oral tongue patients. [file 13046_2015_138_MOESM3_ESM.pdf]

Supporting Information Table 2. Prognostic factors of disease-free survival in oral tongue patients.

	Univariate Analysis	Multivariate Analysis		
	p value	Relative Risk of Death	95% Confident Interval of RR	*p* value
Age (≦50/>50 years)	0.869	0.79	0.20-3.19	0.741
Gender (Female/Male)	0.844	0.7	0.13-3.73	0.676
AJCC Stage (Early/Advanced)	0.025**	25.309	1.52-421.75	0.024**
PNI (-/+)	0.275	0.414	0.09-1.95	0.264
LVP (-/+)	0.208	0.506	0.11-2.42	0.393
ECS (-/+)	0.215	1.477	0.38-5.83	0.577
Radiotherapy (-/+)	0.173	0.731	0.10-5.49	0.761
Chemotherapy (-/+)	0.952	0.156	0.02-1.18	0.072*
IGFBP7 Methylation (-/+)	0.025**	18.282	1.01-332.43	0.05*

PNI: perineural infiltration; LVP: lymphovascular permeation; ECS: extracapsular spread. * $p < 0.1$, ** $p < 0.05$
